# Sun Safety Knowledge, Practices, and Attitudes in the West of Iranian Farmers in 2024: A Cross‐Sectional Study

**DOI:** 10.1002/hsr2.70446

**Published:** 2025-02-13

**Authors:** Farzam Bidarpoor, Halimeh Farazandeh, Payman Ghaderi, Kamal Ebrahimi, Hassan Mahmoodi

**Affiliations:** ^1^ Department of Vice Chancellor for Health Affairs Kurdistan University of Medical Sciences Sanandaj Iran; ^2^ Social Determinants of Health Research Center, Research Institute for Health Development Kurdistan University of Medical Sciences Sanandaj Iran

**Keywords:** attitude, farmer, Iran, knowledge, practice, skin cancer

## Abstract

**Background and Aims:**

Due to the high risk of skin cancer from sun exposure in farmers, understanding their awareness of preventive behaviors is critical. Therefore, this study aimed to determine awareness, attitudes, and practices regarding skin cancer prevention among Iranian farmers.

**Methods:**

This cross‐sectional study was conducted using a valid questionnaire with 336 farmers randomly selected from villages in Kurdistan Province, Iran. To be eligible for the study, participants had to be 18 years of age or older and currently working on a farm in the study region.

**Results:**

The mean age years of participants was 49.31 ± SD = 12.23. The mean ± SD of attitude and performance scores were 11.50 ± 3.78 (min = 2, max = 20) and 9.05 ± 2.48 (min = 0, max = 15), respectively. The findings indicate that while 90.2% of participants are aware of the risks of prolonged sun exposure, misconceptions remain. Only 46.4% recognize UV dangers on cloudy days, and 63.4% understand sunscreen use is needed beyond sunny conditions. Awareness of specific risks like skin cancer (42.6%) and sunburn (27.4%) is moderate, while knowledge of cataracts (6.8%) and premature skin aging (9.2%) is low. Barriers to sunscreen use persist, with only 22.3% deeming it important and 40.8% finding it convenient. The risk of developing skin cancer was underestimated in 131 (39.0%) participants.

**Conclusion:**

Knowledge, attitudes, and practices regarding skin cancer prevention among farmers in Iran are low. There is a need for culturally appropriate educational messages and intervention programs to increase awareness, improve attitudes, and promote preventive behaviors among farmers.

## 1 Background

Cancer is a leading cause of mortality worldwide, accounting for 13% of all global deaths [1]. The number of new cancer cases is expected to increase by about 70% over the next two decades [2]. The incidence of skin cancer, both melanoma and non‐melanoma, has been increasing in recent decades, although most skin cancers are preventable [3]. The main environmental risk factor for skin cancer is prolonged exposure to sunlight [4].

While prolonged sun exposure is the primary risk factor for skin cancer, particularly basal cell carcinoma (BCC), other factors can also contribute to increased risk. For instance, individuals who work outdoors for extended periods, such as farmers and construction workers, have a heightened risk of developing squamous cell carcinoma (SCC) due to chronic sun exposure [5, 6]. Additionally, a family history of skin cancer and a weakened immune system can increase susceptibility to both melanoma and non‐melanoma skin cancers [7, 8].

Farmers, due to their occupational necessity and high exposure to sunlight, are at high risk of developing skin cancer [9]. Most of them understand the risk of developing skin cancer and believe that sun‐protective behaviors reduce the risk of skin cancer, but most farmers do not routinely use adequate sun protection [10, 11].

Studies have shown that farmers either do not have sufficient knowledge about how to protect themselves from harmful environmental factors or do not follow protective measures [12]. Indeed, previous studies from Iran and the USA have shed light on sun exposure and preventive behaviors among farmers. In Iran, findings indicate that nearly half of the farmers (56.4%) have experienced sunburn, with limited use of preventive measures such as sunscreen (8.6%), hats (3.2%), gloves (3.9%), sunglasses (4.6%), and protective clothing (15.4%) [13]. Similarly, in the United States of America, research has shown that less than 50% of farmers adopt sun protection practices, such as wearing wide‐brim hats, long‐sleeve shirts, high‐collared clothing, SPF ≥ 15 sunscreen, or sunglasses [14].

Evidence has shown that women engage in more sun‐protective behaviors than men, including wearing wide‐brimmed hats, sunglasses, long‐sleeved shirts, using sunscreen, and seeking shade [15, 16]. Studies have also shown that knowledge and awareness of ultraviolet radiation exposure and taking sun‐protective measures increase with age, education level, and socioeconomic status [17].

Due to the nature of rural life, sun exposure is more prevalent among rural male farmers, and consequently, they are at a higher risk of developing skin cancer compared to others. This issue is exacerbated when it is estimated that residing in a rural area puts people at a disadvantage in terms of skin cancer survival [18]. Despite efforts to provide more healthcare services to rural areas and consequently reduce the inequality between rural and urban areas, rural residents face more diverse healthcare challenges and problems [19, 20, 21]. One of the most important and fundamental problems in rural areas is the lack of cognitive abilities (such as health knowledge, attitude, and self‐efficacy) and also healthy behaviors [22].

The importance of skin cancer prevention as one of the best strategies in rural areas is highlighted. Therefore, to design educational interventions, it is necessary to assess the level of knowledge, attitude, and practice of farmers regarding skin cancer prevention. Therefore, the aim of this study was to determine the level of knowledge, attitude, and practice of skin cancer prevention among Iranian farmers.

## 2 Methods

This cross‐sectional study was conducted from June 10 to July 25, 2024, in 44 villages from 10 counties in Kurdistan province in the west of Iran. In this study, considering the statistical population of farmers in 10 counties, four health houses were randomly selected from four geographical areas of each county. A “health house” in Iran refers to a primary healthcare facility located in rural areas, where a trained community health worker (CHW) called a “Behvarz” provides basic medical services to villagers, essentially acting as the first point of contact for healthcare within the community; this system is part of Iran's broader primary healthcare network [23]. In proportion to the number of farmers covered by the selected health houses, farmers were also randomly selected in each village. After explaining the study's purpose and obtaining informed consent, Behvarz administered the questionnaire to participants who were illiterate; for literate participants, the questionnaire was self‐administered. Considering the sample size determined for 44 villages, the average number of participants per village was seven to eight people. In case of sample attrition, Behvarz continued with the recruitment process until the number of participants had been reached. This study was approved by the Kurdistan University of Medical Sciences Ethics Committee (Ethics Code: IR.MUK.REC.1402.053). Informed consent was obtained from all participants before their inclusion in the study, ensuring ethical compliance and participant understanding.

### 2.1 Sample Size

Considering the sample size in studies [24], the maximum standard deviation of knowledge about skin cancer protection was equal to 4.5, the acceptable error was 9.0%, the confidence interval was 95%, and the power was 90%. This showed that the minimum required sample size was 138 people. Considering the cluster sampling (two times), the final number of participants in the study was estimated to be 336 people, which includes a 20% sample size drop. *N* =${\;(z1-\alpha /2)}^{2}*\;\delta^{2}$/*d* ^2^

### 2.2 Data Collection Tool

We employed a well‐established instrument to assess knowledge, practices, and attitudes regarding skin cancer among farmers. This instrument has been demonstrated to be reliable and valid [14, 25]. During subsequent evaluation by the research team, discussions and exchange of opinions led to the addition of two questions. These questions address existing attitudes about sunscreen use in the target population of farmers in the covered villages [1]: the belief that sunscreen is only for women and [2] the perception that sunscreen is unpleasant for men. The survey included multiple‐choice questions, Likert scales, and short answer questions. The number of knowledge questions about skin cancer was five items (e.g., “Is prolonged exposure to sunlight harmful?” with response options: Yes = 2, no = 0, and i don't know = 1), the attitude was five items (e.g., “Using sunscreen is important to me,” with a Likert scale ranging from: strongly agree to strongly disagree), and skin cancer prevention behaviors were seven items (e.g., “How often do you use sunscreen (SPF 15/30+) on all exposed skin when working outdoors in sunny weather between 11 a.m. and 3 p.m.?” with response categories: never to always). The original instrument was translated into Farsi (Persian) using a rigorous translation‐back‐translation process. This ensured cultural and linguistic equivalence. While a formal cognitive pretest was not conducted, the questionnaire was piloted with a small sample of farmers to assess its clarity, relevance, and understandability. Feedback from the pilot test was used to refine the questionnaire and ensure its suitability for the target population. The internal consistency of the questionnaire was assessed using Cronbach's *α* coefficient. According to the results, the *α'*s of 0.56, 0.67, and 0.55 for performance, attitude, and awareness are acceptable.

### 2.3 Data Analysis

Descriptive statistics were used to determine the frequency and percentage of qualitative variables (gender, education level, marital status). Analytical statistics were used to determine the mean and standard deviation of quantitative variables such as age, mean knowledge score, attitude score, and performance score. To assess attitude, participants' responses to the attitude items were summed, yielding a score ranging from 0 to 25. Sun safety practices score, ranging from 0 to 20, was similarly calculated based on their responses to the practice questions. Before conducting statistical analyses, normal distribution was tested using the Kolmogorov−Smirnov test. Independent *t*‐tests and ANOVA were used to test the association between attitude, knowledge, and practice score values and covariates. Missing data points were excluded from the analysis. Statistical significance was set at a *p*‐value of less than 0.05.

## 3 Results

A total of 336 people participated with a mean age of 49.31 ± SD = 12.23. The mean (SD) of attitude and performance scores were 11.50 ± 3.78 (min = 2, max = 20) and 9.05 ± 2.48 (min = 0, max = 15), respectively. More information is presented in Table 1.

**Table 1:** Participant demographics.

Variables	Number (%)
Gender	
Male	258 (76.8)
Female	78 (23.2)
Education level	
Illiteracy	209 (62.2)
Primary	69 (20.5)
High school	58 (17.3)
Diploma	
Marital status	
Single	38 (11.3)
Married	298 (88.7)
Sunlight exposure time per day	
Less than half an hour	20 (6.0)
Half an hour to 2 h	48 (14.3)
2−4 h	81 (24.1)
4−6 h	93 (27.7)
6−8 h	94 (28.0)
Number of red or painful sunburns in the past year	
No	59 (17.6)
One time	166 (49.4)
Two times	71 (21.1)
Three times	15 (4.5)
Four times and more	25 (7.4)
Have you ever checked yourself for skin cancer?	
Yes	27 (8.0)
No	309 (92.0)
Have you ever seen a healthcare professional for a skin check?	
Yes	19 (5.7)
No	309 (94.3)

More than two‐thirds of the study participants were male (76.8%). Sixty‐two percent of the participants were illiterate, and 28% spent 6−8 h a day in the sun. Nearly 50% of the participants had at least one episode of red or painful sunburn in the past year. 92% and 94.3% of the participants had never examined themselves for skin cancer and had never visited a healthcare professional for a skin check (Table 1).

Based on Table 2, the findings reveal a high level of awareness regarding the harmful effects of prolonged sun exposure, with 90.2% of participants acknowledging this risk. However, misconceptions persist, as only 46.4% recognize the dangers of UV exposure on cloudy days, and 63.4% understand the need for sunscreen beyond sunny conditions. Awareness of specific risks, such as skin cancer (42.6%) and sunburn (27.4%), is moderate, while knowledge of less recognized effects like cataracts (6.8%) and premature skin aging (9.2%) remains low. Attitudes toward sunscreen usage reflect notable barriers, with only 22.3% considering it important and 40.8% finding it convenient (Table 2).

**Table 2:** Knowledge and attitudes about sun safety amongst farmers in rural west of Iran (*N* = 336).

Knowledge questions	Answered correctly, *N* (%)
Is prolonged exposure to sun harmful?	303 (90.2)
A cloudy day does not protect you from the harmful effects of the sun.	156 (46.4)
You need to use sunscreen only on sunny days	213 (63.4)
Sun exposure is healthy because	
Method of skin tanning	19 (5.7)
Source of vitamin D exposure	196 (58.3)
Helps to improve mood	10 (3.0)
Not healthy at all	111 (33.0)
UV radiation can cause the following	
No health problems	47 (14.0)
Skin cancer	143 (42.6)
Premature skin aging	31 (9.2)
Sunburn	92 (27.4)
Cataracts	23 (6.8)
**Attitude statements**	**Agreed with the statement, *N* (%)**
Using sun protection is important to me	75 (22.3)
Using sun protection is convenient for me	137 (40.8)
I think I am at higher risk of developing skin cancer compared to the average population	131 (39.0)
Sunscreen is only for women	85 (25.3)
It is not graceful for men to use sunscreen	85 (25.3)

Sunscreen reapplication (SPF 15/30+ ) during summer is infrequent, with 53.6% never reapplying and only 11% always doing so. Similarly, the use of sunglasses or UV‐protective glasses is low, with 68.8% never wearing them and only 3.3% always using them. In contrast, wearing regular sunglasses is more common, with 34.2% always and 31.8% often adopting this practice. The use of wide‐brim hats is relatively frequent, as 35.1% always and 27.7% often wear them. Long‐sleeve shirts are highly utilized, with 77.4% always wearing them. Long trousers are the most consistent practice, with 85.1% always using them outdoors. Finally, the use of any sun protection methods for 15 min or more outdoors varies, with 12.8% always and 19% often implementing such strategies. These results suggest a need to improve sunscreen reapplication and UV‐protective practices, emphasizing the importance of comprehensive sun safety measures (Table 3).

**Table 3:** Sun safety practices item by item amongst farmers in rural west of Iran.

Sun safety practice	*N* (%)				
	Always	Often	Sometime	Seldom	Never
How often do you reapply sunscreen (SPF 15/30+) when outdoors in summer (11 a.m.−3 p.m.)?	11 (3.3)	26 (7.7)	58 (17.3)	61 (18.2)	180 (53.6)
How often do you wear sunglasses or glasses with UV protection?	11 (3.3)	20 (6.0)	34 (10.1)	40 (11.9)	231 (68.8)
Wear sunglasses	115 (34.2)	107 (31.8)	71 (21.1)	29 (8.6)	14 (4.2)
Use of a wide brim hat	118 (35.1)	93 (27.7)	76 (22.6)	36 (10.7)	13 (3.9)
Wear a long sleeve shirt	260 (77.4)	62 (18.5)	6 (1.8)	5 (1.5)	3 (0.9)
Wear long trousers	286 (85.1)	32 (9.5)	8 (2.4)	5 (1.5)	5 (1.5)
Frequency of using different methods of sun protection outdoors for 15 min or more^a^	43 (12.8)	64 (19.0)	101 (30.1)	59 (17.6)	69 (20.5)

^a^ Refers to the practice of using multiple sun protection methods simultaneously for at least 15 min. This could include any combination of sunscreen, sunglasses, or glasses with UV protection, a wide‐brim hat, a long‐sleeved shirt, and long trousers.

The analysis highlights variations in sun safety practices and attitudes influenced by demographic and behavioral factors. Gender differences are evident in attitudes, with females scoring higher (mean = 12.85, SD = 3.32) compared to males (mean = 11.09, SD = 3.76). This difference was statistically significant (*p *< 0.001, using *t*‐test). Education level significantly impacts sun safety practices, with diploma holders achieving the highest scores (mean = 18.12, SD = 3.56), suggesting that better education correlates with improved practices (*p *= 0.001, using one‐way ANOVA). Marital status also influences practices, with singles scoring higher (mean = 17.73, SD = 4.52) than others (*p *= 0.038, using *t*‐test). Exposure time to sunlight was associated with better practices among individuals exposed for less than 30 min daily (mean = 18.70, SD = 3.45; *p *= 0.002, using one‐way ANOVA). For attitudes, a significant association was found with the number of sunburns in the past year. Those with no sunburns had higher attitude scores (mean = 12.86, SD = 3.17; *p* = 0.011, using one‐way ANOVA). While performing self‐checks for skin cancer did not significantly influence practices or attitudes, visiting a healthcare professional was associated with higher attitude scores (*p *= 0.028, using *t*‐test) (Table 4).

**Table 4:** Association between participants demographic with attitude and sun safety practices score amongst farmers.

	Attitude		Practice	
Variable	Mean (SD)	*p* value	Mean (SD)	*p* value
Gender				
Male	11.09 (3.76)	> 0.0001	16.30 (3.82)	0.252
Female	12.85 (3.32)		16.97 (4.67)	
Education level				
Illiteracy	11.21 (3.65)	0.068	15.92 (3.88)	< 0.001
Primary	11.53 (3.68)		16.69 (4.48)	
High school				
Diploma	12.50 (3.98)		18.12 (3.56)	
Marital status				
Single	11.92 (4.38)	0.529	17.73 (4.52)	0.038
Married	11.44 (3.65)		16.29 (3.95)	
Sunlight exposure time per day				
Less than half an hour	11.00 (5.59)		18.70 (3.45)	
Half an hour to 2 h	12.60 (2.90)		16.16 (3.72)	
2−4 h	11.71 (3.09)	0.137	16.20 (4.41)	0.002
4−6 h	10.93 (3.78)		16.76 (3.45)	
6−8 h	11.42 (4.03)		16.05 (4.38)	
Number of red or painful sunburns in the past year				
No	12.86 (3.17)		9.11 (1.93)	
One case	10.98 (3.73)		9.03 (2.48)	
Two cases	11.49 (4.12)	0.011	9.78 (2.32)	0.084
Three cases	10.60 (2.94)		8.26 (2.34)	
Four cases and more	12.28 (3.55)		7.40 (3.30)	
Have you ever checked yourself for skin cancer?				
Yes	10.48 (3.75)	0.149	17.59 (3.10)	0.129
No	11.59 (3.73)		16.36 (4.09)	
Have you ever seen a healthcare professional for a skin check?				
Yes	13.21 (3.22)	0.028	17.68 (3.66)	0.174
No	11.40 (3.74)		16.38 (4.05)	

## 4 Discussion

The aim of this study was to determine the knowledge, attitude, and preventive practices of skin cancer among farmers in rural areas of Kurdistan Province. The results of this study showed that 33.3% of farmers answered the knowledge questions correctly, and 30.54% had a positive attitude toward preventive behaviors for skin cancer. 35.8% used all of the explored preventive measures always when working outdoors for skin cancer completely.

A considerable percentage of individuals (92% and 94%) had never examined themselves for skin cancer or visited a dermatologist. This finding is inconsistent with other studies, such as a cross‐sectional study conducted by Fletcher et al., which showed that only 29.4% of farmers sought examination or visits by health professionals for changes in their skin [26]. This is concerning, as skin cancer is one of the most common cancers in the world, and early detection plays a vital role in treatment and recovery. Several factors may contribute to individuals not visiting skin healthcare professionals, including a lack of awareness about the importance of skin checks, financial constraints, geographical distance, or cultural and social factors [27].

The results of our study showed that more than 50% of farmers do not use sunscreen (SPF 15/30+), and nearly two‐thirds do not use sunglasses or sunglasses with UV filters. These findings are consistent with a study by Trenerry et al., which reported that only 16.6% and 10.5% of farmers used sunscreen and sunglasses, respectively [28]. Several factors may explain this low usage. The work environment of farmers, who often spend long hours outdoors exposed to sunlight, might contribute to their lack of sunscreen and sunglass use. While their work environment may provide some natural shade, such as from trees or equipment, this shade may not be sufficient to fully protect against UV exposure. Additionally, many farmers rely on protective clothing, such as long‐sleeved shirts, brimmed hats, and boots, which can provide some sun protection and may be perceived as adequate [29]. Cultural factors may also play a role, as sunscreen and sunglasses might not be considered essential or traditional in some regions, and farmers may prioritize other methods like seeking shade or wearing clothing to reduce exposure [30]. Farmers may rely on other methods, such as protective clothing or seeking shade, to reduce their exposure to sunlight. Moreover, cost and access to sunscreen and sunglasses could be barriers, especially in rural areas where such items may be relatively expensive or unavailable. Farmers with limited financial resources may prioritize agricultural supplies over protective products. Finally, the perceived need for sunscreen and sunglasses may be low among farmers due to limited awareness of the long‐term effects of sun exposure on skin and eye health, a perception influenced by cultural beliefs and a lack of education on the topic [28]. Some farmers may not perceive the need for sunscreen and sunglasses due to various factors, including limited awareness of the long‐term effects of sun exposure on skin health or UV‐related eye conditions. This perception may be influenced by cultural beliefs or a lack of education on the topic [31].

Our study showed that farmers who spent fewer hours per day in the sun had better knowledge, attitudes, and practices regarding skin cancer prevention compared to those who spent more hours per day in the sun. Additionally, individuals who had fewer sunburns in the past year had better knowledge, attitudes, and practices than those who had more sunburns. These results are consistent with a study conducted in Australia, which showed that individuals with no or few sunburns had better practices compared to those with multiple sunburns [25].

The findings of this study also showed that women had better knowledge, attitudes, and practices regarding skin cancer prevention compared to men. This is in line with previous studies that have shown that women generally have better knowledge and practices regarding skin care than men [32]. Gender differences in attitudes toward skin care may stem from personal beliefs and perceptions. Some individuals may hold the belief that engaging in skin care routines is more feminine or unnecessary. On the other hand, some may view skin care as an act of self‐care that contributes to their overall health, regardless of gender. While some studies have found older men to have better skin care practices than women [33], traditional gender roles and societal expectations often shape individuals' attitudes and behaviors toward body care. Historically, there has been a perception that skin care is primarily a female concern, leading to a difference in the level of importance placed on skin care routines between the two genders. Men may feel societal pressure to prioritize other aspects of their appearance or may simply have different cultural norms around skin care.

More than 50% of the individuals considered sunlight as a source of vitamin D. This finding is consistent with a study conducted by D'Souza et al., which showed that over 80% of farmers considered sunlight as a source of vitamin D [25]. There could be several reasons for this finding: Lack of awareness: Farmers may not be aware of the role of sunlight in vitamin D production. Misinformation: Farmers may have received incorrect information about the risks of sunlight and avoid sun exposure to obtain vitamin D. Cultural limitations: Sun exposure may be perceived as an inappropriate practice in the cultural context of the farming community. Occupational constraints: Farmers may not be exposed to enough sunlight for vitamin D production due to long working hours outdoors. Only one‐third of the farmers were aware that sunlight is not always healthy. This finding contradicts the results of a study conducted in Australia, where over 84% of individuals stated that sunlight is not always healthy [25].

The findings of this study suggest that farmers' knowledge about skin cancer prevention may not necessarily translate into changes in their attitudes and practices. Although this study did not specifically test the correlation between knowledge, attitude, and practices, the observed trends are consistent with the results of a study conducted in Australia, which found that skin cancer prevention campaigns, while increasing farmers' knowledge, had a limited impact on their attitudes and practices toward skin cancer prevention. This highlights the potential need for interventions that address barriers beyond knowledge improvement [34]. Despite the increase in awareness about skin cancer, evidence suggests that farmers' attitudes and practices toward prevention have a weak correlation with their knowledge level. This is concerning, as farmers are at risk of developing skin cancer due to their prolonged exposure to sunlight. Possible reasons for the lack of correlation: Economic factors: Many farmers do not have enough time to follow‐up on skin cancer prevention measures due to their busy schedules and long working hours. Cultural barriers: In some cultures, using sunscreen and other preventive measures for skin cancer is perceived as a feminine practice, and men are less likely to do them. Lack of belief in the risk: Some farmers may not be fully aware of the risks of skin cancer or may not take it seriously. Limited access to resources: In some areas, access to sunscreen and other protective products is difficult or expensive [27].

The findings of this study highlight a significant gap in knowledge, attitudes, and preventive behaviors among farmers in rural Kurdistan Province regarding skin cancer. Despite the increasing prevalence of skin cancer globally, our results indicate that farmers in this region are not adequately informed or motivated to protect themselves from sun exposure. Based on these findings, several concrete measures can be implemented to improve skin cancer prevention in this population: Targeted education campaigns: Develop and implement targeted educational campaigns tailored to the specific needs and cultural context of rural farmers. These campaigns should emphasize the importance of sun protection, the risks of skin cancer, and the benefits of early detection. Accessible healthcare services: Increase access to dermatological services in rural areas through mobile clinics or dermatology. This would allow farmers to receive timely skin examinations and advice. Community‐based interventions: Engage with local communities to promote skin cancer prevention through CHW or local leaders. This approach can help to address cultural barriers and increase awareness. Workplace interventions: Collaborate with agricultural organizations to implement workplace sun protection policies, such as providing access to shade, sunscreen, and protective clothing.

The limitations of this study include the unequal sample size of male and female participants, the fact that the majority of the participants were illiterate, and the possibility of recall bias. However, rigorous data quality checks were implemented to identify and address any inconsistencies or errors that may have arisen during the interview process to mitigate. In future research, exploring alternative data collection methods, such as semi‐structured interviews or focus groups, may be considered for illiterate populations to gather richer and more nuanced data.

## 5 Conclusion

The low level of awareness, attitude, and practices of farmers living in rural areas of western Iran, and the lack of correlation between their knowledge and attitude and practices regarding skin cancer prevention, is a serious problem that needs to be effectively addressed by health professionals, including health workers and home healthcare providers in health centers and health posts. By increasing awareness, changing attitudes, and facilitating access to resources, farmers can be helped to protect themselves from skin cancer.

## Author Contributions

**Hassan Mahmoodi:** writing–review and editing, writing–original draft, supervision, methodology, investigation, data curation, conceptualization. **Farzam Bidarpoor:** writing–review and editing, writing–original draft. **Halimeh Farazandeh:** writing–review and editing, data curation. **Payman Ghaderi:** writing–review and editing, methodology. **Kamal Ebrahimi:** writing–review and editing, methodology.

## Conflicts of Interest

The authors declare no conflicts of interest.

## Transparency Statement

The lead author Hassan Mahmoodi affirms that this manuscript is an honest, accurate, and transparent account of the study being reported; that no important aspects of the study have been omitted; and that any discrepancies from the study as planned (and, if relevant, registered) have been explained.

## Data Availability

The data that support the findings of this study are available from the corresponding author upon reasonable request.
